# Life Extension of Aged Jointed Plain Concrete Pavement through Remodeling Index–Based Analysis

**DOI:** 10.3390/ma13132982

**Published:** 2020-07-04

**Authors:** Haekook Jung, Yongjae Kim, Seungwon Kim, Cheolwoo Park, Jeong-Hee Nam

**Affiliations:** 1 Department of Civil Engineering, Kangwon National University, 346 Jungang-ro, Samcheok-si 25913, Korea; jhk-0418@nate.com (H.J.); yzkim@kangwon.ac.kr (Y.K.); 2 KIIT (Kangwon Institute of Inclusive Technology), Kangwon National University, 1 Gangwondaegil, Chuncheon-si 24341, Korea; 3 Department of Infrastructure Safety Research, Korea Institute of Civil Engineering and Building Technology, 283 Goyang-daero, Ilsanseo-gu, Goyang-si 10223, Korea; archnam@kict.re.kr

**Keywords:** jointed plain concrete pavement (JPCP), pavement management system (PMS), remodeling index (RMI), concrete pavement of aging, concrete pavement maintenance, preventive maintenance

## Abstract

As jointed plain concrete pavements (JPCP) age in South Korea, the cost of pavement maintenance is increasing annually. To extend the life of jointed concrete pavements through preventive maintenance, this study used 2017 pavement management system data to analyze the effects of traffic volume, alkali–silica reaction (ASR) grade, age, smoothness, and damaged area on the remodeling index (RMI—a measure of expressway pavement condition). In addition, this study evaluates the final RMI as well as the corresponding pavement condition and change in RMI value after conducting preventive maintenance in lieu of resurfacing or overlaying. The results demonstrated that the effect of ASR grade increased as the RMI forecast year increased and that change in surface distress (△SD) increased with age (most intensively when the pavement was 15–20 years of age). Moreover, change in international roughness index (△IRI) increased with age and traffic volume (similarly within 15–20 years of pavement age). Hence, preventive maintenance is a must for sections with high traffic volume and age even if the RMI is low. Finally, performing repairs through preventive maintenance decreases the number of expressway sections requiring resurfacing and overlaying, thus extending the life of the concrete pavement.

## 1. Introduction

Concrete pavements were first constructed in South Korea in 1987, starting with Expressway 1, and currently account for more than 60% of the expressway network [1]. They have a typical service life of at least 20 years. During 2002–2012, the mileage of aged concrete pavements increased threefold, from 360 to 1150 km [2]. Consequently, in 2016, road pavement maintenance in South Korea cost KRW 770 billion, 2.65 times of that in 2001 (KRW 290 billion). Concrete pavements can be classified as jointed concrete pavements and continuous reinforced concrete pavements, which in South Korea accounted for 9491 lane-km (97%) and 245 lane-km (3%), respectively, in 2015 [3,4]. A large proportion of jointed concrete pavements are on expressways, and their maintenance required KRW 130 billion in 2014, 3.84 times of that in 2001 (KRW 35 billion) [1,5]. Clearly, pavement maintenance costs are increasing annually due to aging-related damage. Thus, to control costs, performing appropriate maintenance by predicting concrete pavement life depending on current conditions is imperative [3]. Researches show that more than half of the concrete pavement gone through the partial depth repair would be damage again within 10 years. The insufficient analysis of the problem, the use of unsuitable repair materials the adoption of inappropriate design methodology and the poor quality of the construction may be the reasons for this problem [6]. If the roads are not properly maintained, roads will deteriorate with time and significant cost will have to be incurred to make repairs. A well-designed pavement is likely to stay in good condition for a long time, and pavement life can be significantly increased by performing preventative maintenance [7]. The combined impact of traffic loads and climate are constantly deteriorating highway pavements. The current situation and amount of deterioration is defined as the ability to explain the effects of deterioration caused by traffic and climate during design life [8]. The remodeling index (RMI), a measure of expressway pavement condition, developed in 2017, is a measure of the pavement condition and is based on the aging of concrete pavements on expressways managed by the Korea Expressway Corporation. The RMI is used to identify continuous sections of pavement that require resurfacing or overlay (O/L). The RMI prediction model was developed using multi-regression analysis of the correlation among the RMIs of the representative sections evaluated using the panel rating and their influential factors [5]. The independent variables of the prediction model, such as the pavement conditions, pavement age, traffic volume, and weather conditions were used to analyze the RMI sensitivity [5]. The sensitivity of change in surface distress (ΔSD) was affected following the order of annual range of temperature, international roughness index (IRI), surface distress (SD), and annually consumed amount of deicing salts and of change in IRI (ΔIRI) was affected following the order of pavement age, annual average daily traffic (AADT), IRI, alkali-silica reaction (ASR) grade, annual range of temperature, and number of days with precipitation [1,9,10].

Pavement condition index (PCI) was used to give a score of pavement functional condition and to enhance the decision-making process for Kazakhstan [9]. Many countries’ experiences confirm that proper management of road infrastructure must rely on detailed and up-to-date information on the condition of a pavement’s surface [11,12,13,14]. The PCI is an objective-type method for visually evaluating pavements, which considers the extent and severity of the defects found in a particular study site. This method was applied in many research papers [15,16,17,18,19]. The PCI can be determined more quickly and simply through subjective evaluations. The use of the GIS (Geographic Information System) tool facilitated the visualization of the pavement conditions in the different sections [20]. In small-scale repairs, secondary distress frequently occurs in the repaired part because the road should be opened to traffic as soon as possible and, as a result, a sufficient time required for repair and curing cannot be assured [21]. Because frequent traffic congestion due to small-scale repair works generates additional social expenses, large-scale repair works (remodeling works) of deteriorated concrete pavement sections with long extension need to be performed to dramatically improve the overall pavement conditions and lower maintenance costs [22]. The present study examined the feasibility of extending the life of jointed concrete pavements by identifying changes in the RMI score when conducting preventive maintenance (diamond grinding, cross-section repair, and crack sealing) in lieu of O/L and resurfacing. To this end, this study uses the 2017 pavement management system (PMS) data of National Expressway 2 and data from the Korea Meteorological Administration of the Ministry of Land, Infrastructure and Transport [3] to analyze the effects of ASR grade, age, forecast year, and traffic volume on the RMI. The results indicated that timely and appropriate preventive maintenance, primarily based on the RMI, ASR, age, and traffic, decreases the number of expressway sections requiring resurfacing and overlaying, thus extending the life of the concrete pavement; in addition, the longer maintenance is delayed, the higher the maintenance cost.

## 2. RMI (Remodeling Index) System

### 2.1. RMI (Remodeling Index)

Composed of RMI is in research paper [5]. Because aged concrete pavement cannot be improved at once, a RMI can evaluate future pavement condition by evaluating the current status of aged concrete pavement and allows the selection of improvement priority sections and repair methods according to the pavement condition. The composition of RMI can be found in the research paper [5].

The RMI can be used to estimate the deterioration in pavement condition over time. Measures in the RMI system include the “current RMI” and the “change in RMI” (△RMI) over 1 year and N years in the future [5].

RMI = RMIpresent + N × △RMI(1)

### 2.2. RMIpresent (Remodeling Index Present)

Current RMI represents the current pavement condition and comprises SD and IRI [5].

RMIpresent = 1.688 × log (1 + 2 × SD) + 1.74 × IRI(2)

### 2.3. △RMI (△Remodeling Index)

△RMI represents the change in RMI over a 1-year period and is obtained by extrapolating the current RMI considering the corresponding change in SD and IRI (i.e., △SD and △IRI) [5].

△RMI = 1.688 × log (1 + 2 × △SD) + 1.74 × △IRI(3)

### 2.4. △SD (△Surface Distress) and △IRI(△International Roughness Index)

△SD and △IRI is representative of IRI, SD, AADT, annual range temperature, service life, use of snow removal agent, ASR grade, and number of days of precipitation [5].

△SD = EXP (−12.15 + 0.56 × IRI + 0.083 × SD + 0.0077 × use of snow removal agent + 0.2 × annual range temperature)(4)

△IRI = (−0.43 + 0.024 × IRI + 0.00014 × service life2 + 0.0039 × AADT + 0.017 × ASR grade + 0.00333 × annual range + 0.002 × number of days of precipitation)(5)

## 3. RMI (Remodeling Index) Factor Analysis

All analyses in this study were applied to the Seoul-bound Lane 1 of National Expressway 1. A RMI of one to five indicates relatively good concrete condition, a RMI of five to seven indicates the need for O/L or regular repair due to partial damage, and a RMI of seven or higher indicates the need for O/L or resurfacing (i.e., remodeling) due to extensive cracks or re-damage after cross-sectional repairs.

### 3.1. RMI (Remodeling Index) by ASR (Alkali-Silica Reaction) Grade

**Figure 1:**
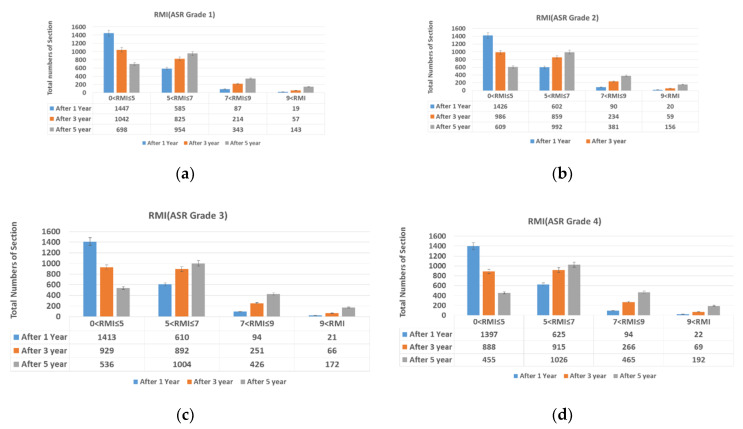
Total number of sections by ASR grade and RMI (**a**) ASR grade 1; (**b**) ASR grade 2; (**c**) ASR grade 3; and (**d**) ASR grade 4.

This study examined the change in the RMI value of sections of the Seoul-bound Lane 1 of National Expressway 1 1, 3, and 5 years into the future depending on the current ASR grade (Figure 1). Table 1 shows the criteria for RMI.

Table 2 shows the ASR is classified into four grades ASR grade one, ASR grade two, ASR grade three, and ASR grade four. High ASR grade has the high risk for the damage of pavement. Table 2 show the damage caused by ASR in concrete pavement with respect with ASR grade.

Table 3 shows the ASR grade and number of RMI sections 1, 3, and 5 years into the future. Clearly, the higher the ASR grade and the further into the future the forecast year, the higher the number of target sections identified as requiring remodeling. In other words, the longer maintenance is delayed, the higher the maintenance cost.

Considering an ASR grade of one, 1 year into the future, 1447 sections were of RMI one to five, 585 were of RMI five to seven, and 106 were of RMI or higher. Considering the worst ASR grade (ASR four), 1397 sections were of RMI one to five, 625 were of RMI five to seven, and 116 were of RMI or higher. Thus, as the ASR grade increased, so did the number of sections whose RMI was or higher.

In the 1-year RMI projections, the number of sections graded RMI or higher differed by 10 between ASR one and ASR four. In the 5-year projections, this difference was 171. That is, the wider the difference in the ASR grade and the further into the future the forecast year N, the greater the difference in the number of sections graded RMI or higher. Section with a higher ASR grade can change RMI rating followed by years and it should be repaired at an appropriate time. If delayed, the time for maintenance O/L level repair is necessary.

### 3.2. △IRI (△International Roughness Index) by Section and ASR (Alkali-Silica Reaction) Grade

**Figure 2:**
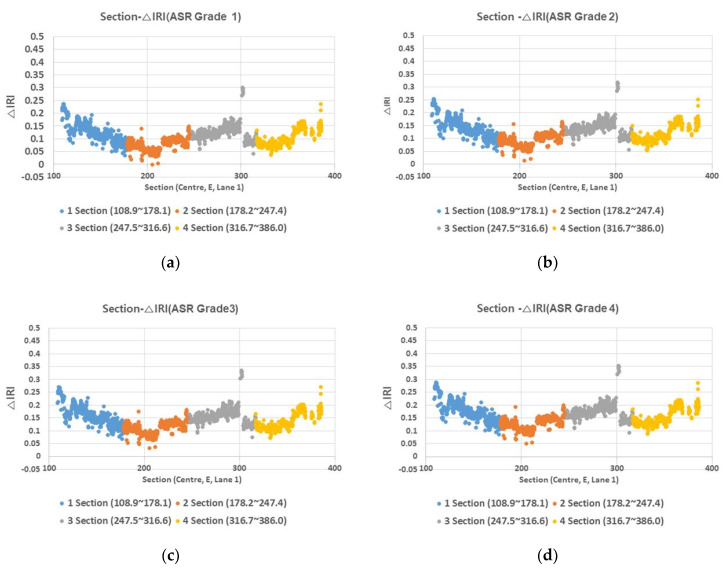
Section-change in international roughness index (△IRI) (ASR grade) relationship. (**a**) ASR grade 1; (**b**) ASR grade 2; (**c**) ASR grade 3; and (**d**) ASR grade 4.

This study examined △IRI with respect to ASR grade for the Seoul-bound Lane 1 of Expressway 1. In the analysis, the expressway was divided into four sections, and the effect of ASR grade (ASR one to four) on △IRI was studied (Figure 2).

**Figure 3:**
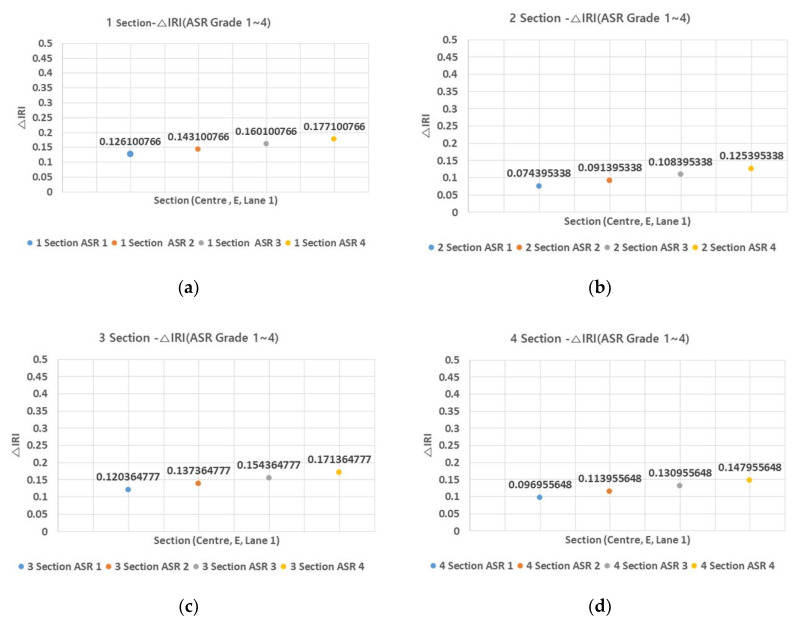
Section–ΔIRI relationship (ASR grade 1–4) (**a**) 1 section; (**b**) 2 section; (**c**) 3 section; and (**d**) 4 section.

**Figure 4:**
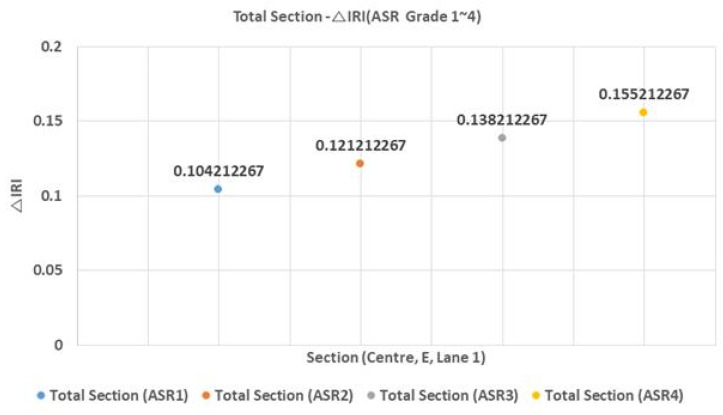
Total section–ΔIRI relationship (ASR grade 1–4).

Under different ASR grades, △IRI varied only by about 0.1, indicating that the ASR grade has a negligible effect on △IRI. Figure 3 and Figure 4 show the average △IRI by ASR grade for the four sections and for all sections, respectively.

△IRI varied by an average of 0.05 between ASR one and ASR four, further evidencing the weak effect of ASR grade on △IRI. However, with an increase in the predicted year N, the impact of the ASR grade is expected to increase, and the higher section of the ASR grade is expected to require appropriate maintenance.

### 3.3. △RMI (△ Remodeling Index) by ASR (Alkali-Silica Reaction) Grade

**Figure 5:**
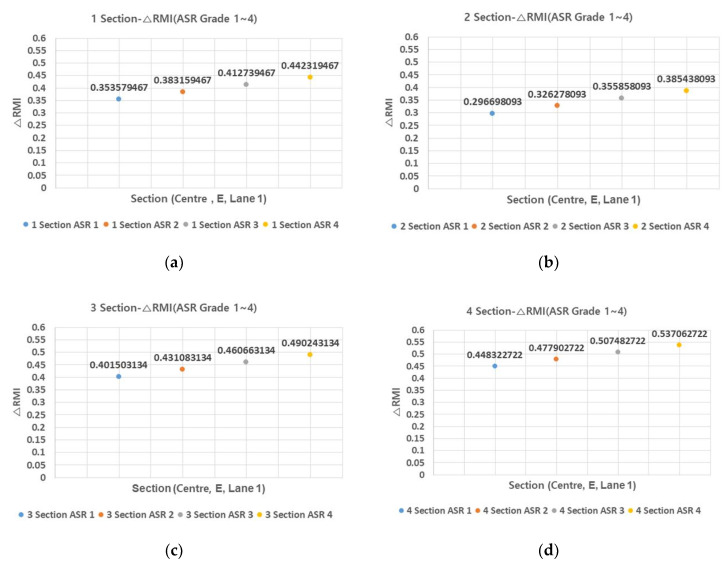
Section–ΔRMI relationship (ASR grade 1–4) (**a**) 1 section; (**b**) 2 section; (**c**) 3 section; and (**d**) 4 section.

**Figure 6:**
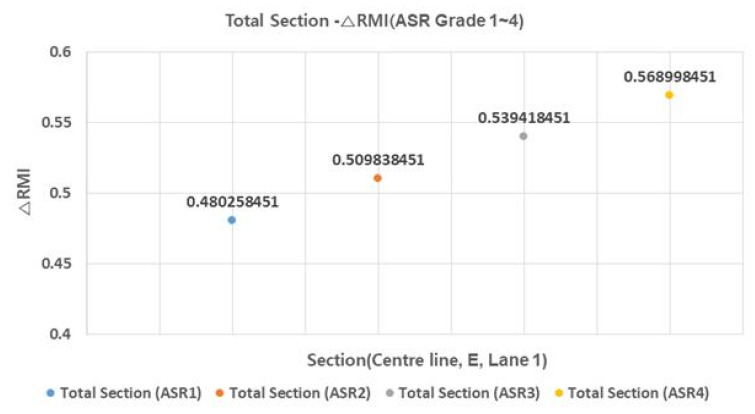
Total section–ΔRMI relationship (ASR grade 1–4).

△RMI comprises △SD and △IRI, and △IRI varies depending on the ASR grade. Figure 5 and Figure 6 show the average △RMI by ASR grade for the four sections and for all sections, respectively.

In △RMI, the difference between ASR grade one and grade four is about 0.9. This is found to have a greater impact than the difference between ASR grade one and ASR grade four of △IRI. As for ASR grade, the impact is considered to be higher than the final RMI, so it is necessary to review proper repair measures according to ASR grade.

### 3.4. Relationship of △IRI (△International Roughness Index) with Traffic Volume and Pavement Age

**Figure 7:**
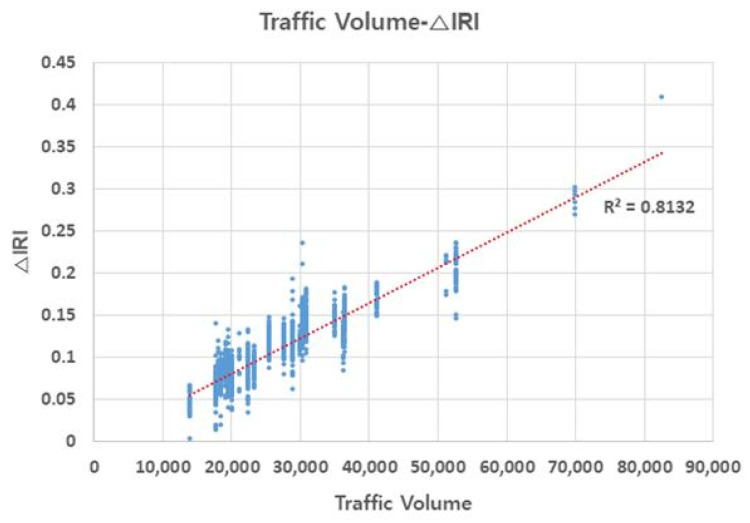
Traffic volume–ΔIRI relationship.

**Figure 8:**
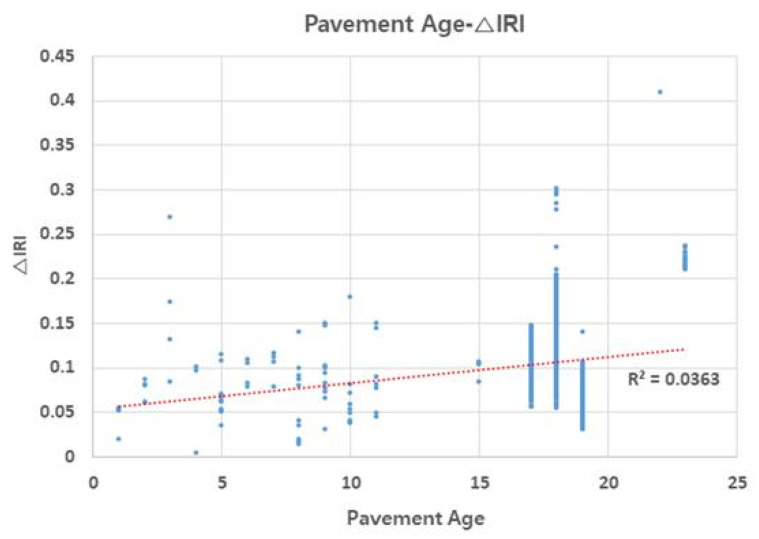
Pavement age–ΔIRI relationship.

Deterministic IRI performance models include the accumulated heavy traffic as a variable for increasing the roughness as heavy vehicles are said to be responsible for pavement damaging, due to its higher weight, implying a greater load over the pavement [23]. Figure 7 and Figure 8 illustrate the relationship of △IRI with traffic volume and pavement age (which both affect △IRI), respectively. Pavement age is the time elapsed since it was poured in place and left to set. △IRI tended to increase with increase in traffic volume, showing high accuracy. Moreover, △IRI tended to increase with pavement age, but most of the change occurred when the pavement was 15–20 years of age. Furthermore, sections with healthy pavement showed little change in △IRI. These results suggest that in addition to the appropriate repair measures for sections with high RMI, sections with high traffic volume and pavement age require preventive maintenance even if their RMI is low.

### 3.5. Effect of Pavement Age on △SD (△Surface Distress)

**Figure 9:**
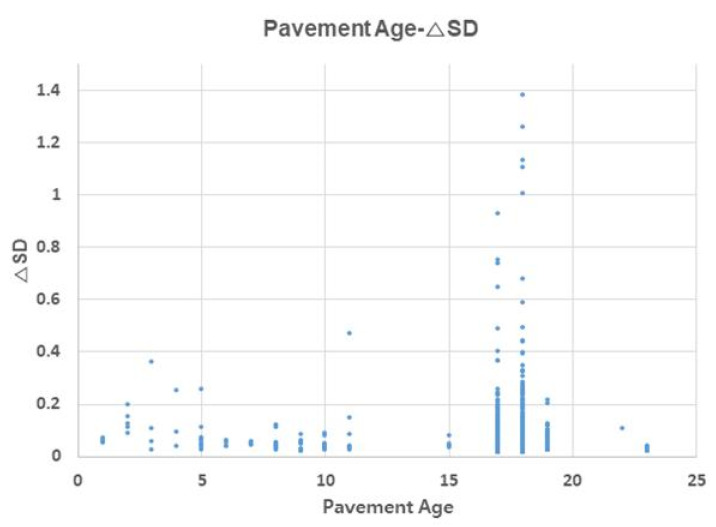
Pavement age–ΔSD relationship.

Figure 9 shows the effect of pavement age on △SD. As pavement age increases, △SD tended to increase, but this trend was concentrated within 15–20 years of age, and △SD changed little in sections with healthy pavement. Thus, concrete pavements undergo intensive damage after 15 years of age. To increase the life span of the pavement, sections of 15 years or more pavement age required periodic maintenance measures.

### 3.6. Effect of Forecast Year N and ASR (Alkali-Silica Reaction) on RMI (Remodeling Index)

**Figure 10:**
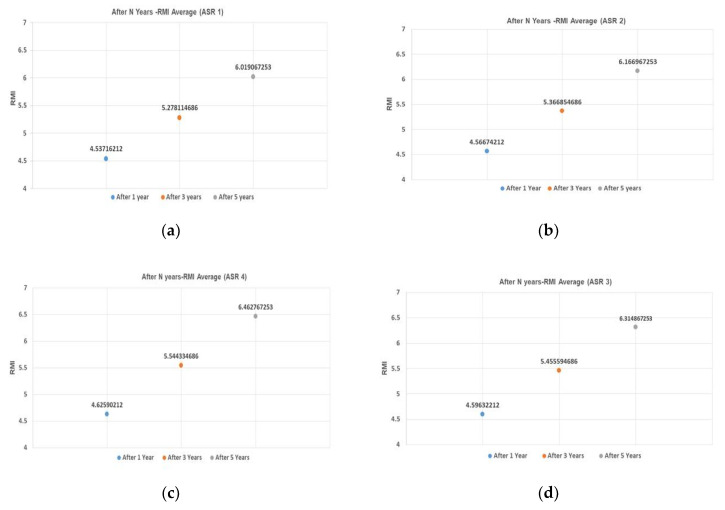
RMI average and ASR grades and after N-years into the future. (**a**)ASR grade 1; (**b**) ASR grade 2; (**c**) ASR grade 3; and (**d**) ASR grade 4.

Notwithstanding the weak effect of ASR grade on RMI, this study found that the further into the future is the forecast year N, the higher the increase in RMI. This study verified this relationship for forecasts 1, 3, and 5 years into the future. Figure 10 shows the RMI average for four ASR grades 1, 3, and 5 years into the future. In the 1-year forecasts, RMI differed by approximately 0.09 between ASR one and ASR four, and the corresponding difference was approximately 0.44 in the 5-year forecasts. Thus, the effect of ASR grade on RMI was intensified with increase in the forecast year N. Hence, sections with a high ASR grade must undergo preventive maintenance even if their RMI is low. In addition, when RMI is high, timely O/L is crucial as deterioration accelerates with delay in repair.

## 4. Applying Preventive Maintenance on the Basis of RMI (Remodeling Index)

According to the criteria for section remodeling used by the Korea Expressway Corporation, resurfacing or O/L of sections is recommended when the RMI is seven or higher, provided that the section lengths add up to at least 3.0 km, with continuous homogeneity. Regular maintenance or O/L is recommended for sections with RMI five to seven, and no repair measures are recommended for sections with RMI five or lower.

This study examined changes in RMI after preventive maintenance on sections where remodeling was deemed not urgent according to the foregoing criteria. RMI was analyzed on the basis of SD and IRI in terms of change in pavement condition.

### 4.1. IRI (International Roughness Index)-Based Preventive Maintenance

**Figure 11:**
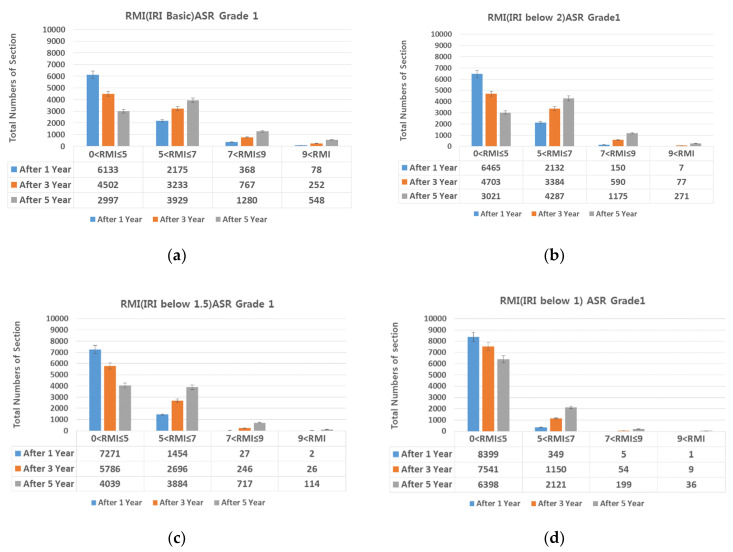
Change in number of sections per RMI with respect to IRI. (**a**) basic IRI and ASR grade 1; (**b**) IRI 2.0 and ASR grade 1; (**c**) IRI 1.5 and ASR grade 1; and (**d**) IRI 1 and ASR grade 1.

**Figure 12:**
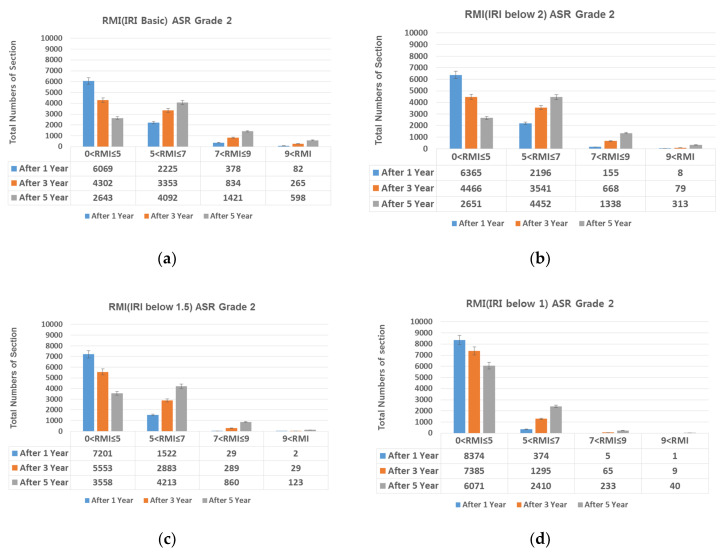
Change in number of sections per RMI with respect to IRI. (**a**) basic IRI and ASR grade 2; (**b**) IRI 2.0 and ASR grade 2; (**c**) IRI 1.5 and ASR grade 2; and (**d**) IRI 1 and ASR grade 2.

**Figure 13:**
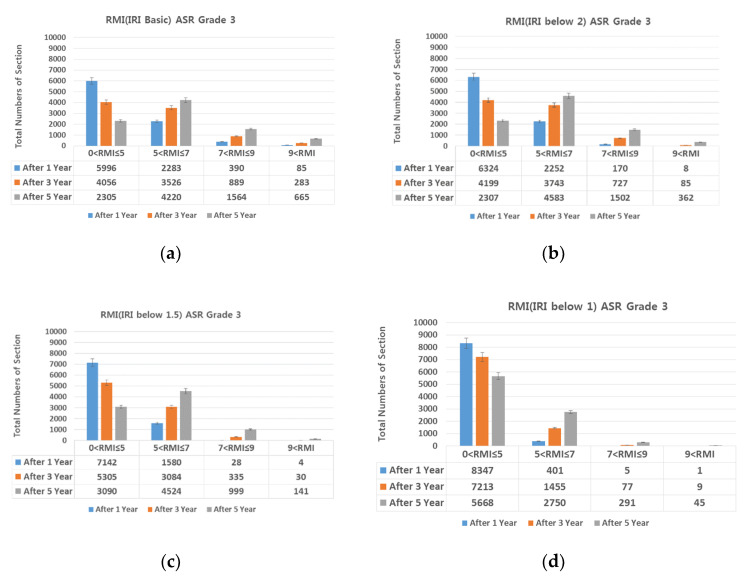
Change in number of sections per RMI with respect to IRI. (**a**) basic IRI and ASR grade 3; (**b**) IRI 2.0 and ASR grade 3; (**c**) IRI 1.5 and ASR grade 3; and (**d**) IRI 1 and ASR grade 3.

**Figure 14:**
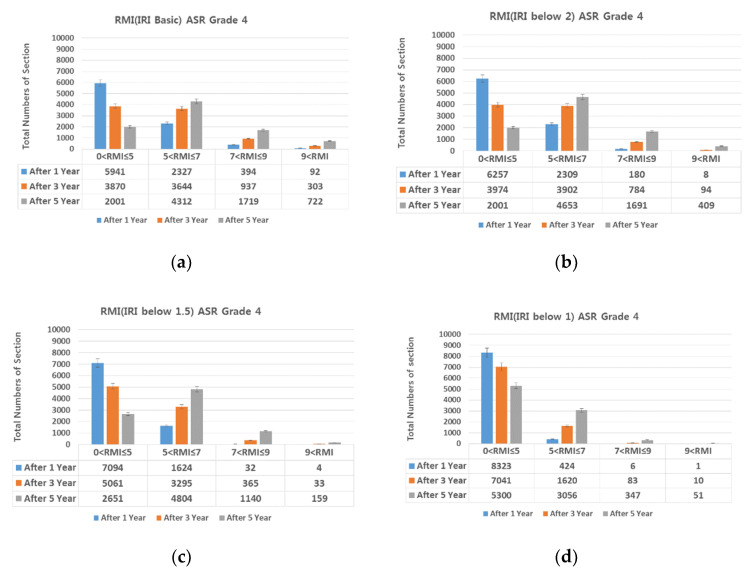
Change in number of sections per RMI with respect to IRI. (**a**) basic IRI and ASR grade 4; (**b**) IRI 2.0 and ASR grade 4; (**c**) IRI 1.5 and ASR grade 4; and (**d**) IRI 1 and ASR grade 4.

Diamond grinding was selected as the preventive maintenance measure for achieving smoothness. On diamond grinding, the IRI decreased 1.5 or lower. For Lanes 1, 2, and 3 of Expressway 1 in both directions, RMI under different ASR grades assuming IRI of 2.0, 1.5, and 1.0 was compared with RMI after 1, 3, and 5 years assuming no repair (Figure 11, Figure 12, Figure 13 and Figure 14).

Provided diamond grinding decreases IRI, the number of sections with RMI one to five (good pavement condition) also increases. At an IRI of 2.0 or lower, although the number of sections with RMI one to five increases, the increase is smaller than when no repair is conducted. At IRI 1.5–1.0 and lower, the number of sections with RMI one to five increased sharply. Approximately 96%, 86%, and 73% of the analyzed sections were RMI one to five at ASR one and IRI under 1.0 (the best pavement condition in the analyzed data) after 1, 3, and 5 years, respectively. On the other hand, for RMI one to five at ASR four and baseline IRI (no repair)—the worst pavement condition in this study—the corresponding values were approximately 68%, 44%, and 23%. While IRI is the conventional measure of roughness and should be reduced after diamond grinding. On the other hand, if diamond grinding is not done correctly, a poor pavement performance can be expected [24].

In summary, diamond grinding decreases the number of sections that require resurfacing and O/L. Hence, to extend the life of the pavement through preventive maintenance, an IRI of 1.5 or lower must be realized through diamond grinding.

### 4.2. SD (Surface Distress)-Based Preventive Maintenance

**Figure 15:**
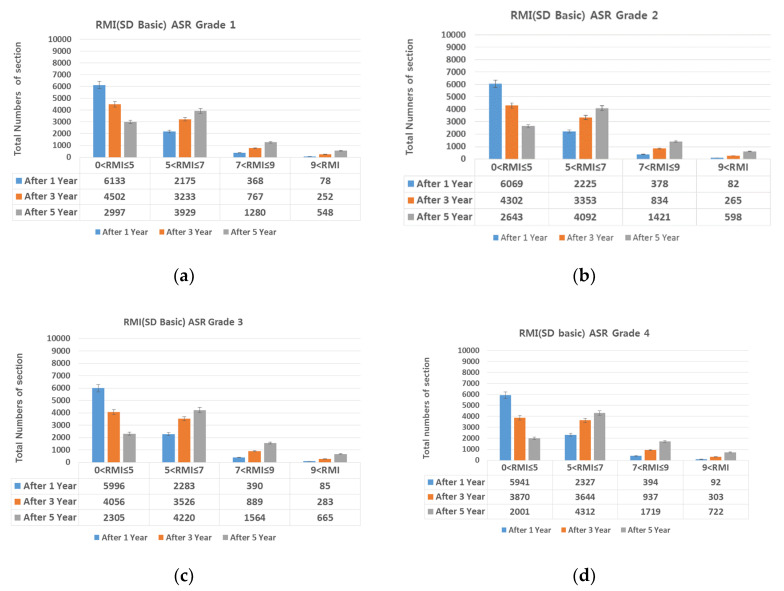
Change in number of sections per RMI with respect to surface distress (SD). (**a**) basic SD and ASR grade 1; (**b**) basic SD and ASR grade 2; (**c**) basic SD and ASR grade 3; and (**d**) basic SD and ASR grade 4.

**Figure 16:**
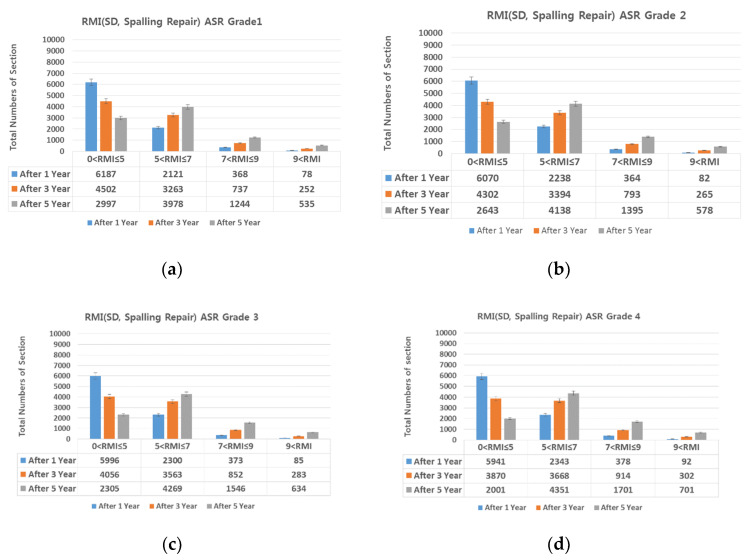
(**a**) spalling repair and ASR grade 1; (**b**) spalling repair and ASR grade 2; (**c**) spalling repair and ASR grade 3; and (**d**) spalling repair and ASR grade 4.

**Figure 17:**
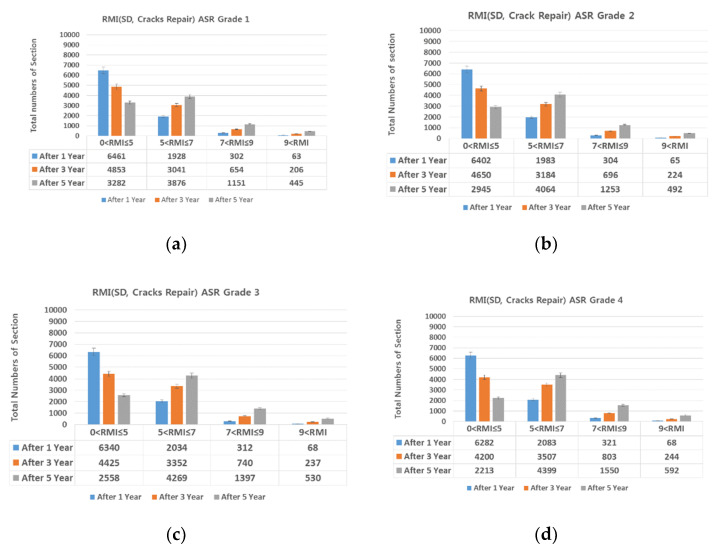
(**a**) crack repair and ASR grade 1; (**b**) crack repair and ASR grade 2; (**c**) crack repair and ASR grade 3; and (**d**) crack repair and ASR grade 4.

**Figure 18:**
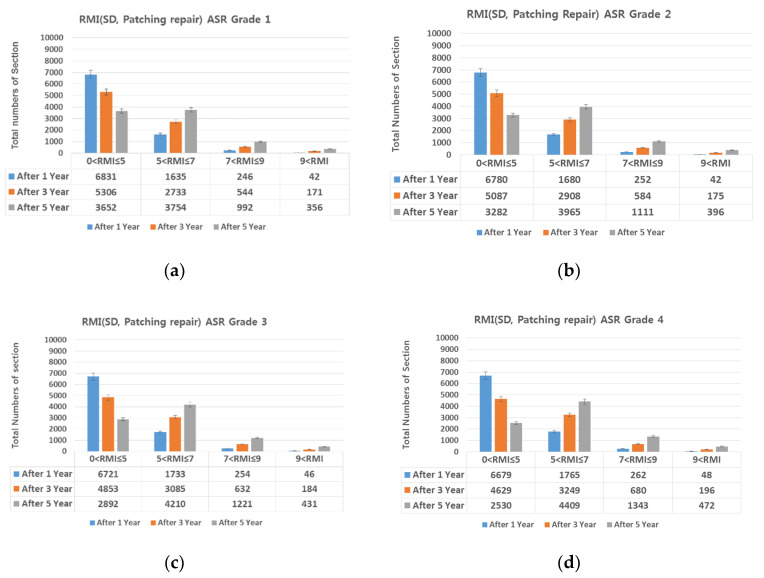
Change in number of sections per RMI with respect to SD. (**a**) patching repair and ASR grade 1; (**b**) patching repair and ASR grade 2; (**c**) patching repair and ASR grade 3; and (**d**) patching repair and ASR grade 4.

**Figure 19:**
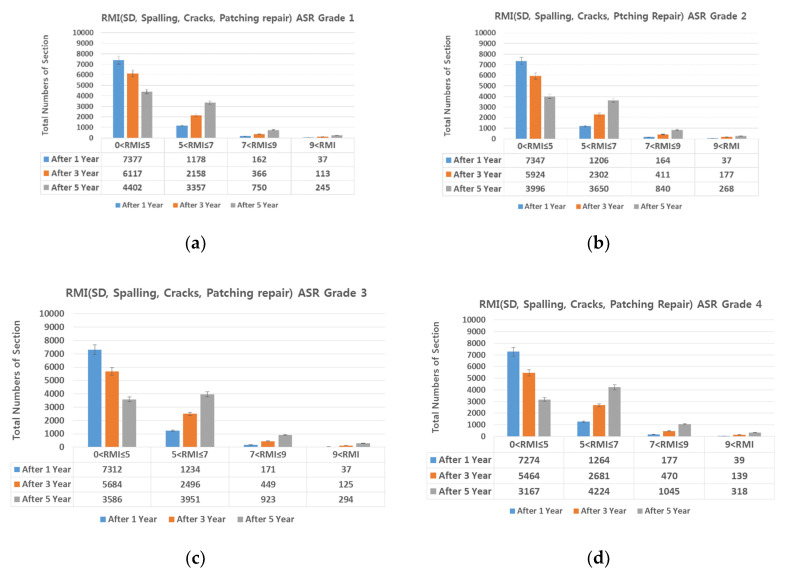
Change in number of sections per RMI with respect to SD. (**a**) spalling, cracks, patching repair, and ASR grade 1; (**b**) spalling, cracks, patching repair, and ASR grade 2; (**c**) spalling, cracks, patching repair, and ASR grade 3; and (**d**) spalling, cracks, patching repair, and ASR grade 4.

The proportions of damage types on all expressways (linear cracks 22.52%, surface cracks 29.61%, alligator cracks 0.46%, patching 43.29%, and spalling 4.12%). The SD damaged area was divided by the ratio of each damage type; then, the damage types that could be prevented through maintenance (i.e., linear cracks (crack sealing) and patching and spalling (cross-section repair) were selected, and the SD damaged area was reduced proportional to the repairable area. Figure 15, Figure 16, Figure 17, Figure 18 and Figure 19 show the distribution of sections by RMI grade after 1, 3, and 5 years and by ASR grade of SD after cross-section repair and crack sealing.

On reducing the SD damaged area as described above, the number of sections with RMI one to five (good pavement condition) increased in the following order: Patching, linear cracking, and spalling. For all three damage types, SD decreased by up to 70% after repair. For RMI of one to five at ASR one assuming all three types of damage were repaired, 84%, 70%, and 50% of the sections had a good RMI after 1, 3, and 5 years, whereas the corresponding values at ASR four were 68%, 44%, and 23%. That is, as the ASR grade worsens, the proportion of the pavement in good condition decreases. However, this is favorable for extending the life of the pavement when performing preventive maintenance based on damage type as in research papers [6,7]. In addition, repairing damage through a combination of repair measures is more advantageous than through a single measure.

### 4.3. SD (Surface Distress) and IRI (International Roughness Index)-Based Preventive Maintenance

Compared with the reduction in SD damaged area due to repair, after applying cross-section repair and crack sealing to prevent spalling, linear cracks, and patching and applying diamond grinding to secure IR smoothness, relatively more sections were of RMI one to five, indicating good pavement conditions with smoothness of IRI 1.5 or lower. This indicates that IRI smoothness repair is more advantageous than SD damage repair in terms of preventive maintenance. However, SD damage repair and IRI smoothness repair can be implemented together to further extend the service life of concrete pavements.

## 5. Conclusions

This study analyzed the effect of factors applied in RMI using the 2017 PMS data of Expressway 1, examined changes in RMI score when preventive maintenance is applied to jointed concrete pavement rather than O/L or resurfacing, and confirmed the extension in life of the aged jointed concrete pavement. The conclusions are as follows:

1. The higher the ASR grade and the further into the future the forecast year, the higher the number of target sections for remodeling. The more maintenance is delayed, the higher the maintenance cost.
2. Although the ASR grade did not substantially affect △IRI, the effect of ASR is expected to increase with increase in the RMI forecast year N.
3. Regarding the effect of ASR on RMI, the range of increase according to ASR grade was larger than the change in △IRI, and the impact of △IRI was greater than △SD.
4. As traffic volume and pavement age increased, △IRI tended to increase, but this increase was concentrated within 15–20 years of pavement age. Therefore, in sections with high traffic volume and pavement age, preventive maintenance is a must even if the RMI is low.
5. As pavement age increased, △SD tended to increase, but this increase was concentrated within 15–20 years of pavement age. That is, △SD changed little in sections with healthy pavement, but the concrete pavement underwent intensive damage after 15 years of age.
6. The effect of ASR grade on RMI increased with increase in the forecast year. Therefore, preventive maintenance is a must for sections with a high ASR grade even when the RMI is low. In addition, when the RMI is high, timely resurfacing and O/L are essential as deterioration accelerates with delay in repair.
7. Diamond grinding decreases the number of sections requiring resurfacing and O/L. Thus, to extend the life of the pavement, diamond grinding can be used as a preventive maintenance to achieve an IRI of 1.5 or lower.
8. Smoothness repair is more advantageous than damage repair in terms of preventive maintenance. However, both SD damage repair and IRI smoothness repair can be implemented together to extend the service life of concrete pavements.

## Figures and Tables

**Table 1:** Remodeling index (RMI) urgency criteria [5].

RMI	Remodeling Urgency	Judgment Criteria	Recommended Repair Method
9 < RMI	Urgent	With severe cracking, severe re-damage of partial/full depth repair	Re-paving or overlay
7 < RMI ≤ 9	Necessary	With considerable cracking, Considerable re-damage of partial/full depth repair	
5 < RMI ≤ 7	Considerable	In a comparatively moderate condition	Typical repair or overlay
0 < RMI ≤ 5	Unnecessary	-	-

**Table 2:** The damage caused by alkali-silica reaction (ASR) in concrete slab with respect to ASR grade.

ASR Grade	Appearance of Concrete Slab	Damage Slab Area (%)	Crack Width (mm)	Crack Spacing (mm)
1	-Some cracks and some reticular cracks occur in the slab	1–9	<0.5	>1000
	-Check for signs of alkali-aggregate reaction (core collection)			
2	-Attack cracks and reticular cracks in slabs	10–24	0.5–1	500–1000
	-Pop-out and spalling do not occur			
	-Some horizontal cracks in the concrete pavement (core collection)			
3	-Attack cracks and reticular cracks in slabs	25–49	1–5	100–500
	-Surface leakage of reactive gel			
	-Some pop-out and spalling occur			
	-A large number of heat generated inside the concrete slab (core collection)			
4	-Attack cracks and reticular cracks in slabs	More then 50	>5	<100
	-Surface leakage of reactive gel			
	-Pop-out and spalling multiple occurrences			
	-Horizontal pupil inside the concrete slab (core collection)			

**Table 3:** ASR grade and number of RMI sections 1, 3, and 5 years into the future.

ASR Grade	Years	RMI Sections			
		0 < RMI ≤ 5	5 < RMI ≤ 7	7 < RMI ≤ 9	9 < RMI
ASR 1 (Total number of sections)	After 1 year	1447	585	87	19
	After 3 years	1042	858	214	57
	After 5 years	698	954	343	143
ASR 2 (Total number of sections)	After 1 year	1426	602	90	20
	After 3 years	986	859	234	59
	After 5 years	609	992	381	156
ASR 3 (Total number of sections)	After 1 year	1413	610	94	21
	After 3 years	929	892	251	66
	After 5 years	536	1004	426	172
ASR 4 (Total number of sections)	After 1 year	1397	625	94	22
	After 3 years	888	915	266	69
	After 5 years	455	1026	465	192
